# Dr. Wm. Pepper’s Case of Aneurism of the Thoracic Aorta, Opening into the Left Bronchus; with Remarks

**Published:** 1843-01-21

**Authors:** Wm. Pepper

**Affiliations:** Physician to the Pennsylvania Hospital, &c. &c.


### CASE OF ANEURISM OF THE THORACIC AORTA,

*Opening into the Left Bronchus; with Remarks.*

BY WM. PEPPER, M. D.,

Physician to the Pennsylvania Hospital, &c. &c.

[*Read before the Philadelphia Pathological Society, December* 26, 1842.]

Rudolph S———, a seaman, aged about 35 years, entered the Pennsylvania Hospital, May 25th, 1842.

He stated that in the early part of last winter he had been bled and blistered for severe pain of the left side; and that since then he has had cough and difficulty of breathing. About two weeks before his admission these symptoms had increased, and he was obliged to abandon his occupation.

*Present Condition.*—Pulse 85, full and regular; slight cough, and expectoration of puriform matter; tongue clean; temperature natural; no emaciation—at the summit of the left lung the respiration and percussion are perfectly natural; but throughout the rest of this lung there is no trace of vesicular murmur, and over the same extent, the percussion is perfectly flat; at the root of the lung there is bronchial respiration, and resonance of the voice.

Nothing unusual on the right side, excepting slight bronchial respiration at the root of the lung.

The sounds of the heart can be heard throughout the left side, but, in other respects, they are perfectly natural.

There is no difference in the mensuration of the two sides of the thorax.

During the first week of his residence in the Hospital, the patient used small doses of morphia, and a restricted diet, at the end of which time, six drops of Lugol's iodine solution were administered three times a-day, and the following ointment was applied over the left side:

Iodin., ℈j.

Iodide Potass., ℈ij.

Cerat. Simp., ℥j.

This treatment was continued up to June 30th; and, excepting an occasional paroxysm of hectic fever, there was no important change in the symptoms. From this time up to July 25th, he was under the care of Dr. Stewardson; at the end of this period, there was no perceptible change in the health of the patient, nor was there any decided emaciation.

He was now using iodid. potass., gr. v. three times a-day; and a seton was introduced on the left side of the thorax.

On the morning of July 31st, he was walking about, and appeared in all respects as well as usual; the same evening, during a paroxysm of cough, he expired suddenly, with profuse hæmorrhage from the mouth and nose.

*Autopsy the following day.*

The left lung, excepting a small portion of the upper lobe, was perfectly indurated; when incised, it presented a smooth surface, of a grey colour, and slightly tinged with pink; the upper part of the lung contained no tubercles, and was perfectly healthy; bronchial tubes dilated, and filled with fluid blood; pleura much thickened, and adherent throughout.

The entire substance of the right lung presented the appearance of pulmonary apoplexy, and the bronchial tubes were filled with florid blood.

The aorta, where it crosses the left bronchus, was much dilated, and communicated with the latter by an ulcerated opening about one-quarter of an inch in diameter; for several lines around the perforation, the mucous membrane of the bronchus was inflamed, and a small portion of it formed a species of valve to the opening.

The dilated portion of the aorta extended from the third to the fifth dorsal vertebra; its upper part was about two inches in diameter, gradually diminishing as it descended. The aneurism contained no coagula; and its inner coat was much thickened and corrugated; above and below the tumour, the aorta was perfectly healthy, as were also its valves. Heart natural. Other organs not examined.

*Remarks.*

The morbid appearances, in the above case, are particularly interesting. The left lung presented a rare example of chronic pneumonia, differing in many particulars from simple tubercular infiltration; independent of its peculiar appearance, the fact that there were no tubercles in the summit of the lung, nor in the lung of the opposite side, would, of itself be sufficient to show that the above lesion had a different origin. This opinion is confirmed by the circumstance, that the patient had, some six months previous, an acute disease of the left lung; from which time, up to his death, he had some cough and dyspnœa, but his general health had suffered comparatively little, as is frequently the case in chronic pneumonia; whereas, in tubercular infiltration, the constitutional irritation and the emaciation would have been much greater.

It is highly probable that the appearance presented by the right lung occurred but a few moments previous to death; for in the morning the respiration on the right side was perfectly natural, and the patient had no increase of dyspnœa, or other symptoms during the day. After that, a direct communication was established between the bronchiæ and the aorta; the violent efforts at inspiration may have forced the arterial blood into the minute bronchial tubes and air vesicles, thus producing somewhat the appearance of pulmonary apoplexy.

The existence of the aneurism was not suspected during the life of the patient; the pulsations heard at the root of the lung were sufficiently explained by the indurated lung conducting the sounds of the heart; and this condition also accounted for the bronchial respiration. Had the lung been perfectly healthy, the strong pulsations and bronchial respiration, heard at its root, would have been sufficient to have indicated the existence of the aneurism, or, at least, to have induced such a suspicion. In chronic pneumonia the bronchial tubes are generally dilated, as in the above instance; and, under ordinary circumstances, bronchial respiration and resonance of the voice can be heard throughout the indurated lung.

The pressure of the aneurism on the left bronchus must have impeded the free access of air, and, therefore, explains the local character of the physical signs in this case.

May not the long continued pressure of the indurated lung upon the thoracic aorta, have induced chronic inflammation of that vessel, followed by dilatation and ulceration of its different coats?

